# Experimental Study on Creep–Recovery Behavior of Polyphosphoric Acid (PPA) Modified Asphalt Binders under Multiple Factors

**DOI:** 10.3390/ma16072740

**Published:** 2023-03-29

**Authors:** Shuangquan Jiang, Xiuming Jiang, Huifeng Li, Zhan Ding, Peilong Li, Mingkai Zhou

**Affiliations:** 1 School of Highway, Chang’an University, Xi’an 710064, China; 2 Sichuan Road and Bridge (Group) Co., Ltd., Chengdu 610041, China; 3 Key Laboratory of Road Structure & Material Ministry of Transport, Chang’an University, Xi’an 710064, China; 4 School of Water and Environment, Chang’an University, Xi’an 710064, China

**Keywords:** polyphosphoric acid (PPA) modified asphalt, multiple factors, creep–recovery behavior, rheology simulation, energy parameters

## Abstract

The polyphosphoric acid (PPA) modified asphalt binder is a potential choice as one of the pavement materials for its excellent high-temperature performance and low cost. To further analyze the influences of temperature and load on the service life of pavement from the perspective of deformation behavior, six kinds of asphalt binders with different PPA dosages were prepared for Multiple Stress Creep and Recovery (MSCR) tests at five temperature levels. The deformation behavior is investigated by basic deformation parameters, rheological simulation, and energy parameter changes. The results show that the percent recovery (R) drops sharply while non-recoverable creep compliance ($J_{nr})$ goes up slightly with the increase in temperature. Three-element model, composed by $E_{1}$, $\eta_{1}$, and $\eta_{2}$, can be used to describe the creep behavior. PPA-modified asphalt binder exhibits nonlinear creep behavior, and the logarithmic model can simulate recovery behavior better than the power–law model. Stored energy and dissipated energy can characterize the change of energy in the creep process under different conditions and show a significant correlation to deformation parameters. It is concluded that the elastic component of asphalt binders is increased by PPA, which is beneficial to the improvement of the deformation resistance and recovery capacity of asphalt binders. The recommended dosage of PPA is 1.5%. This investigation is conducive to a better understanding of the deformation behavior of PPA-modified asphalt binders and provides a reference for its engineering applications.

## 1. Introduction

The asphalt binder is a typical viscoelastic material [1] whose mechanical behavior and deformation characteristics have temperature and time dependence [2]. The deformation of asphalt binder caused by load can only partially recover, and the unrecoverable part will accumulate under repeated load, resulting in high-temperature deformation disease of asphalt pavement [3,4,5]. Therefore, the method of improving the ability of deformation resistance and prolonging the service life of asphalt pavement has been focused on for a long time [3,6]. In addition, polymer (e.g., styrene-butadiene-styrene, polyethylene, rubber, etc.) modified asphalt binders were usually adopted to strengthen the deformation resistance and recovery ability of the asphalt binder [7,8,9,10]. However, polymer-modified asphalts are usually plagued by segregation problems [11], compatibility problems [12,13], high costs, and increased mixing time and temperature, which may lead to the aging of the asphalt binder [14].

In the past few years, as a chemical modifier, polyphosphoric acid (PPA) has gradually come into the vision of researchers [10,15,16]. Pei et al. [17] demonstrated that the compound modification of OSDOA/PPA dramatically enhanced the deformation resistance of the SBS-modified asphalt binder and reduced its low-temperature cracking resistance. Yang et al. [18] prepared composite-modified asphalt with PPA and styrene-butadiene-styrene (SBS) and found that PPA contributed to the formation of the network structure and improved the elastic behavior and high-temperature stability. Wang et al. [19] reported that the addition of PPA can effectively improve the high and low-temperature performance of asphalt binders. Zhang et al. [20] found that the amount of SBS in high-viscosity asphalt binders can be reduced by adding PPA, and it showed positive effects on physical and rheological properties. Li et al. [21] proved that, with an increase in PPA dosage, the rutting resistance of asphalt binders was dramatically improved, and the recommended amount of PPA addition was 1.6%. Similar findings can be found in the investigation of Wei et al. [22]. Reactions of grafting, phosphate esterification, and cyclization are triggered in asphalt binders with the addition of PPA, which is considered as a cause of changing its carbon chain structure [23,24]. Thus, the amount of macromolecular substances in asphalt binders is increased to improve the high-temperature performance [25].

Since the emergence of PPA-modified asphalt binder, its high-temperature performance has been the focus of researchers [10,15,23,24]. The PPA-modified asphalt binder shows excellent properties such as high-temperature stability [26], stable storage, anti-aging, and anti-fatigue [27], and its application in road engineering is receiving more and more attention [28]. Creep–recovery test and multiple stress creep recovery (MSCR) tests are effective means to evaluate the high-temperature performance of asphalt binders or mixtures [5,29,30,31], in which deformation parameters (e.g., strain recovery rate and residual strain) are normally used [32,33]. Deformation parameters are ordinarily influenced by test conditions (temperature, time, and load level) due to the properties of viscoelastic materials. A full understanding of these influences is conducive to high-temperature performance characterization. However, deformation parameters are the specific description of the experimental phenomenon, and it is almost impossible to reveal the cause from a theoretical perspective. Therefore, many scholars introduce rheological models into the deformation analysis of asphalts or mixtures [34,35]. Li et al. [31] analyzed the viscoelastic response and creep mechanism of asphalt mixtures using the Burgers model. In order to evaluate the low-temperature properties of asphalt binders, including PPA-modified asphalt binder, Aflaki et al. [32] analyzed the bending beam rheometer (BBR) test results by the Burgers model. To obtain more consistent analysis results with actual experimental data, the Burgers model was revised, or other rheological models were adopted. Saboo et al. [33,36] used the power–law model and generalized Burgers model successively to describe the creep and recovery behavior of asphalt binders. Celauro et al. [37] fitted the creep–recovery curves of asphalt mixtures with the fractional derivative form of the Burgers model, and accurate fitting results were obtained. Hajikarimi et al. [38] discussed the creep and stress relaxation behaviors of modified asphalt binders using the generalized fractional nonlinear viscoelastic model. In general, deformation characterization and viscoelastic analysis based on a rheological model are the main research contents of high-temperature creep performance, while the latter pays more attention to revealing creep properties from the theoretical level.

At present, the high-temperature deformation behavior of PPA-modified asphalt binder is mainly evaluated by complex shear modulus ($G^{*}$), rutting factor ($G^{*}/\sin\delta$), and MSCR tests [15,18,23,24]. However, these indicators are usually tested in the linear viscoelastic range of asphalt binders, which is inconsistent with the fact that the rutting mainly occurs in the nonlinear viscoelastic range [39,40,41]. Many researchers have also reported that the correlation between the rutting factor and pavement rutting depth is not strong enough, and the MSCR test is considered a substitute [42]. Typically, MSCR tests are carried out to obtain deformation parameters to evaluate the high-temperature rutting resistance of asphalt binders [43,44,45,46]. Fewer studies have focused on the deformation behavior of asphalt binders from an energy perspective. The creep–recovery properties and nonlinear creep behavior of PPA-modified asphalt binders need to be further analyzed.

In this investigation, creep–recovery curves of PPA-modified asphalt binder at different temperatures were obtained by MSCR test, from which deformation parameters and rheological parameters were acquired. Steady-state creep rate and energy changes during the creep process were further calculated, based on which creep mechanism was revealed from macro-deformation and energy perspectives. It is hoped that the findings can provide references for the evaluation of high-temperature deformation performance and application of PPA-modified asphalt binders.

## 2. Materials and Methods

### 2.1. Asphalt Binders

The neat asphalt binder selected in tests is Shell 90# [47], and the modifier PPA is analytically pure, whose P_2_O_5_ content is above 85%. PPA-modified asphalt binder was prepared by stirring at 600–700 rpm for 40 min under 160–165 °C, and PPA dosage, calculated by mass of the neat asphalt binders, was 0%, 0.5%, 1.0%, 1.5%, 2.0%, and 2.5%, respectively. The basic physical properties of six kinds of asphalt binders are shown in Table 1.

### 2.2. Creep–Recovery Test

According to ASTM D7405-15 [51], the MSCR test was carried out using DHR-I hybrid rheometer (produced by American TA Instruments) at 0.1 kPa and 3.2 kPa. The plate clamp selected for tests has a diameter of 25 mm and a gap of 1 mm. Further, 20 creep–recovery cycles (for N=1 to 20) were run at 0.1 kPa followed by 10 cycles (for N=21 to 30) at 3.2 kPa. For each cycle, creep for 1 s and recover for 9 s, which took a total of 300 s. To analyze the response of deformation behavior to temperature, tests were carried out at 34, 46, 58, 64, and 76 °C.

### 2.3. Methodologies

**Figure 1:**
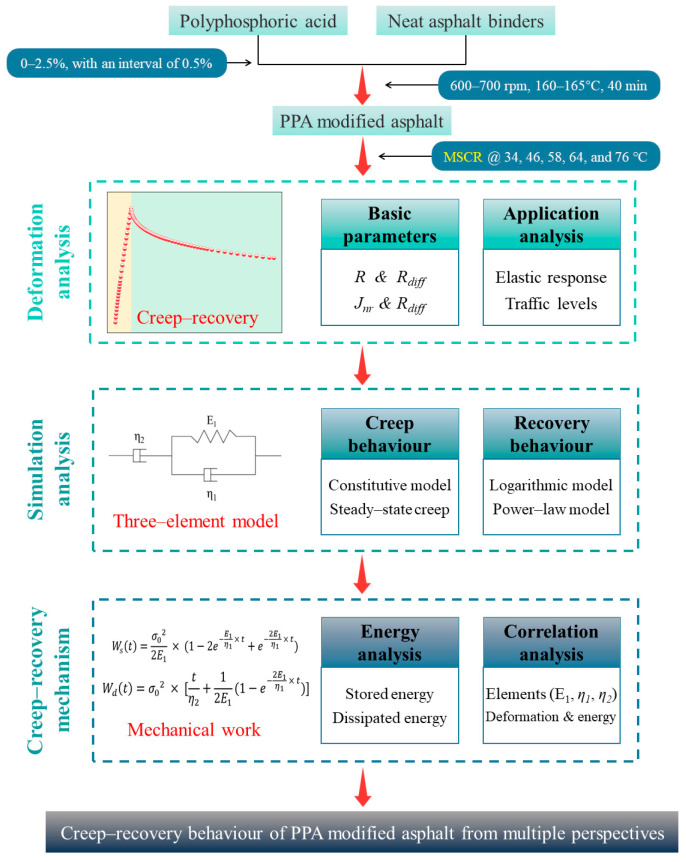
Scheme of the investigation.

To explore the creep–recovery behavior of PPA-modified asphalt binders thoroughly, basic deformation parameters, rheological constitutive model, and energy parameters were introduced into this investigation, as detailed in Figure 1.

#### 2.3.1. Deformation Parameters

**Figure 2:**
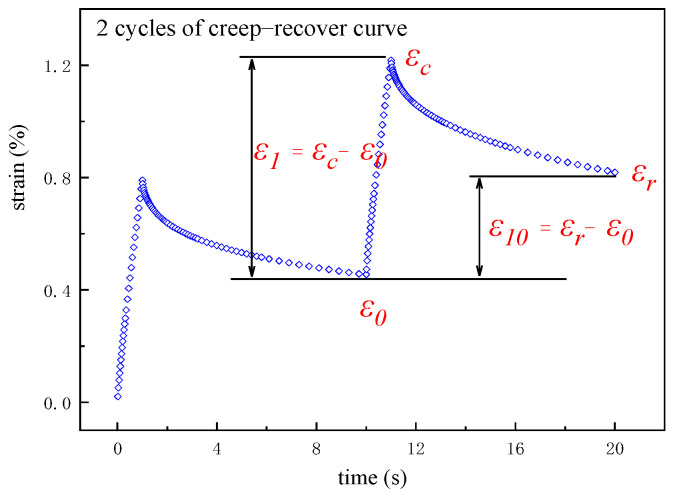
Strain values in the creep−recovery curves.

The creep–recovery curve of asphalt binder is shown in Figure 2.

In Figure 2, $\varepsilon_{0}$ is the initial strain value at the beginning of the creep portion of each cycle, %; $\varepsilon_{c}$ is the strain value at the end of the creep portion of each cycle, %; $\varepsilon_{r}$ is the strain value at the end of the recovery portion of each cycle, %; the adjusted strain value at the end of the creep portion and the recovery portion of each cycle were, respectively, calculated by $\varepsilon_{1}=\varepsilon_{c}-\varepsilon_{0}$ and $\varepsilon_{10}=\varepsilon_{r}-\varepsilon_{0}$.

The average percent recovery (R) and the average non-recoverable creep compliance ($J_{nr}$) have a stable correlation with high-temperature performance in the actual situation, while percent difference in R and $J_{nr}$ between 0.1 kPa and 3.2 kPa show the sensitivity of asphalt binders to stress [32,33]. According to ASTM D7405-15 [51], these parameters can be calculated by Equations (1)–(6):

(1) $$R\left(0.1\right)=\frac{1}{10}\times \sum_{N=11}^{N=20}(\frac{\varepsilon_{1,N}-\varepsilon_{10,N}}{\varepsilon_{1,N}})\times 100$$

(2) $$R\left(3.2\right)=\frac{1}{10}\times \sum_{N=21}^{N=30}(\frac{\varepsilon_{1,N}-\varepsilon_{10,N}}{\varepsilon_{1,N}})\times 100$$

(3) $$J_{nr}\left(0.1\right)=\frac{1}{10}\times \sum_{N=11}^{N=20}\frac{\varepsilon_{10,N}}{0.1}$$

(4) $$J_{nr}\left(3.2\right)=\frac{1}{10}\times \sum_{N=21}^{N=30}\frac{\varepsilon_{10,N}}{3.2}$$

(5) $$R_{diff}=\frac{R\left(0.1\right)-R\left(3.2\right)}{R\left(0.1\right)}\times 100$$

(6) $$J_{nrdiff}=\frac{J_{nr}\left(3.2\right)-J_{nr}\left(0.1\right)}{J_{nr}\left(0.1\right)}\times 100$$

where the units of R and $J_{nr}$ are %, kPa^−1^, respectively; N is the number of creep–recovery cycles. If $(\varepsilon_{1,N}-\varepsilon_{10,N})/\varepsilon_{1,N}<0$ then record it as 0, and calculate $J_{nr,N}$ as $\varepsilon_{1,N}/0.1$ or $\varepsilon_{1,N}/3.2$.

Based on the results of $J_{nr}\left(3.2\right)$, standard (S), high (H), very high (V), and extremely high (E) traffic loading levels are identified in AASHTO M 332-18 [52], as shown in Table 2.

#### 2.3.2. Rheological Model

**Figure 3:**
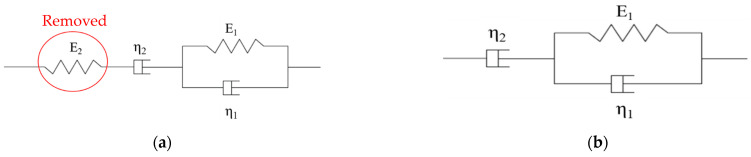
Rheological models: (**a**) Burgers model; (**b**) three-element model.

The rheological model is an idealized analysis method for the mechanical and deformation properties of materials [53,54]. The Burgers model is widely used in describing the deformation characteristics of asphalt binders and asphalt mixtures [31,32], as shown in Figure 3a. However, a preliminary attempt showed that the creep behavior of PPA-modified asphalt binders was not well described by the Burgers model. Therefore, the three-element model [55] ignored $E_{2}$ of Burgers model was selected, as shown in Figure 3b.

In Figure 3, $E_{1}$ and $\eta_{1}$ from Kelvin model, Pa and Pa·s; $E_{2}$ is an elastic element representing the instantaneous elastic deformation, Pa; $\eta_{2}$ is a single viscous element, Pa·s. Thus, the creep stage of PPA-modified asphalt binders can be described by:

(7) $$\varepsilon \left(t\right)=\frac{\sigma_{0}}{E_{1}}\times \left(1-e^{-\frac{E_{1}}{\eta_{1}}\times t}\right)+\frac{\sigma_{0}}{\eta_{2}}\times t$$

If Boltzmann superposition principle applies, the recovery stage can be described as:

(8) $$\varepsilon \left(t\right)=\frac{\sigma_{0}}{E_{1}}\times \left(1-e^{-\frac{E_{1}}{\eta_{1}}\times t_{1}}\right)\times e^{-\frac{E_{1}}{\eta_{1}}\times (t-t_{1})}+\frac{\sigma_{0}}{\eta_{2}}\times t_{1}$$

where t is testing time, s; and unloading at $t_{1}$; $\varepsilon \left(t\right)$ is the strain at time t, %; $\sigma_{0}$ is applied load, 100 Pa or 3200 Pa in this paper.

#### 2.3.3. Energy Calculation

The creep behavior of asphalt binders under external force involves the transformation of energy. In rheological models, $E_{1}$ is an energy storage element, while $\eta_{1}$ and $\eta_{2}$ are energy-consuming components. According to the definition of stress and strain, the energy conversion per unit volume in the creep process of asphalt binders under constant stress can be calculated by the definition of mechanical work (force times path) [56,57]; however, the Burgers model is applied in the literature; therefore, equations are revised as follows:

(9) $$W_{s}\left(t\right)=\frac{\sigma_{0}{}^{2}}{2E_{1}}\times \left(1-2e^{-\frac{E_{1}}{\eta_{1}}\times t}+e^{-\frac{2E_{1}}{\eta_{1}}\times t}\right)$$

(10) $$W_{d}\left(t\right)=\sigma_{0}{}^{2}\times \left[\frac{t}{\eta_{2}}+\frac{1}{2E_{1}}\left(1-e^{-\frac{2E_{1}}{\eta_{1}}\times t}\right)\right]$$

where $W_{s}\left(t\right)$ is stored energy, Pa; $W_{d}\left(t\right)$ is dissipated energy, Pa.

## 3. Results and Discussion

### 3.1. Deformation Parameters Analysis

#### 3.1.1. Variation of R and $J_{nr}$

**Figure 4:**
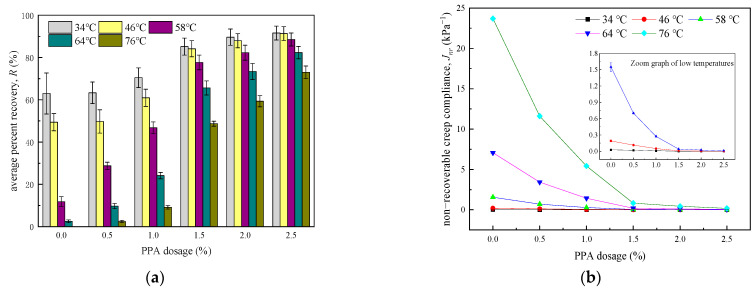

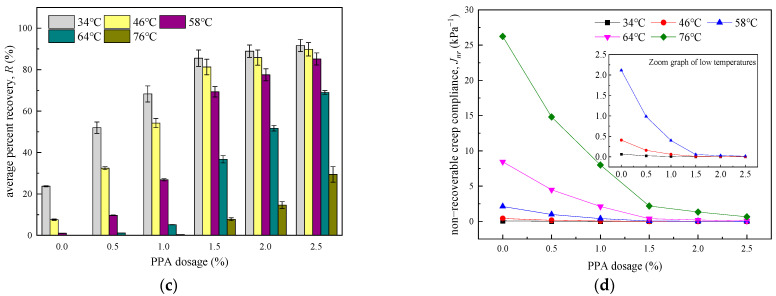
Calculation results of R and $J_{nr}$ (**a**) $R\left(0.1\right)$ (**b**) $J_{nr}\left(0.1\right)$ (**c**) $R\left(3.2\right)$ (**d**) $J_{nr}\left(3.2\right)$.

R and $J_{nr}$ of six kinds of asphalt binders are calculated by Equations (1)–(4), and the results are shown in Figure 4.

It can be seen from Figure 4a that R goes up slightly after a significant increase when PPA is continuously added, and 1.5% is the key dosage. Taking $R\left(0.1\right)$ at 58 °C as an example, the increments are 555.2% and 14.0%, respectively, when the PPA dosage varies from 0% to 1.5% and 1.5% to 2.5%. Further, R drops sharply with the rising temperature, especially when the temperature exceeds 58 °C. Remarkably, four bars of R are missing in the case of high temperature and low PPA dosage, which is caused by the continued development of deformation after unloading. As illustrated by Figure 4b,d, $J_{nr}$ curves go down with the rise in PPA dosage, while $J_{nr}$ is positively correlated with temperature. The results clearly show that $J_{nr}$ is very close to 0 at 34 °C and 46 °C or when the PPA dosage is above 1.5%. In these cases, asphalt binders behave more likely as an elastic material, and usually do not suffer from rutting problems.

The deformation behavior of PPA−modified asphalt binders is related to PPA dosage, temperature, and stress level. There will be a reaction between PPA and neat asphalt binders, so the content of heavy components in asphalt binders and the degree of crosslinking between asphalt molecules will be improved, and as a result, a more stable structure is formed [23,24]. It is also proved by the penetration index in Table 1 that the colloidal structure gradually changes from sol-gel type to gel type [58] when PPA dosage exceeds 1.5%. Thereby, the anti-deformation and recovery ability of asphalt binders can be improved. However, the elevated temperature causes the degradation of heavy components, which leads to asphalt binders converting to a viscous flow state. In addition, this effect is only apparent at more than 46 °C, which may be correlated to the softening point of the neat asphalt binder.

#### 3.1.2. Application Traffic Levels

**Figure 5:**
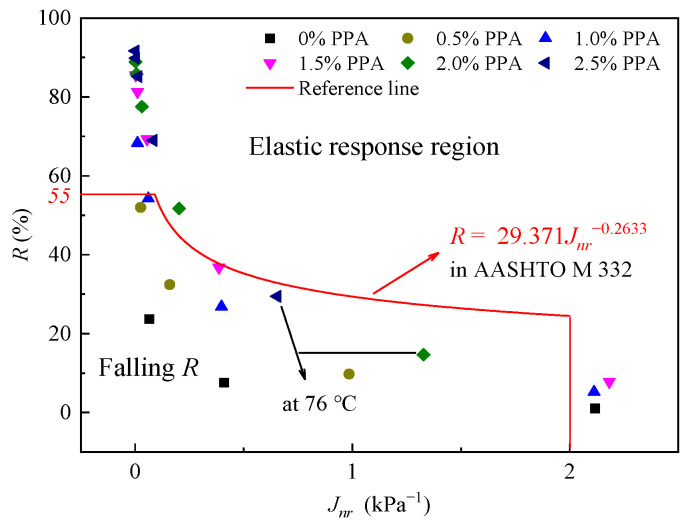
Comparison of $J_{nr}\left(3.2\right)$ and $R\left(3.2\right)$ to assess elastic response.

PPA-modified asphalt binders have greater application value in heavy load and high-temperature areas [5]. The process of detecting the elastic behavior of asphalt binders is accomplished by evaluating $R\left(3.2\right)$ along with the $J_{nr}\left(3.2\right)$, as shown in Figure 5.

The plotted points on or above the line indicate a more pronounced elastic response of asphalt binders, while the points that fall below the line represent a poor elastic recovery ability [42,49]. It can be inferred from Figure 5 that PPA is not a polymer but can still increase the elastic composition of asphalt binders (especially with a dosage higher than 1.5%), which is due to the aforementioned chemical modification. It should be noted that two points at 76 °C fell below the reference line, which may be caused by the degradation of heavy components.

**Figure 6:**
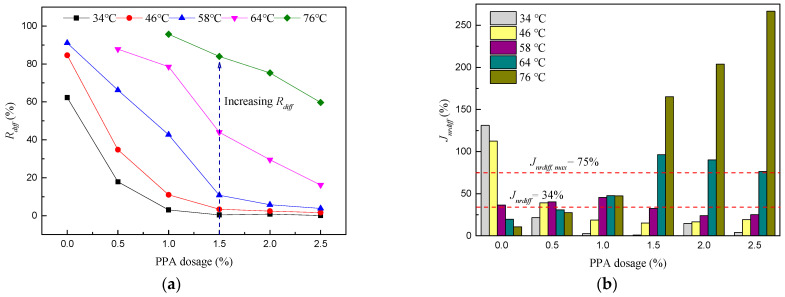
Percent difference in R and $J_{nr}$ between 0.1 kPa and 3.2 kPa (**a**) $R_{diff}$, (**b**) $J_{nrdiff}$.

Figure 6 shows the variation of $R_{diff}$ and $J_{nrdiff}$, calculated by Equations (5) and (6). The neat asphalt binders show the biggest $R_{diff}$ values (if existing) at the same temperature, indicating PPA improves stability against varied stress loading conditions. Meanwhile, an increase in temperature will strengthen the effect of stress on the percent recovery, which is more obvious when the temperature exceeds 46 °C. Compared with $R_{diff}$, the change of $J_{nrdiff}$ is relatively complex and does not show obvious regularity. $J_{nrdiff}\;$floats up and down around 34%, with a STDEV of 8.5 at 58 °C. Note that not all $J_{nrdiff}$ values are below the limiting value (75%) set by AASHTO M 332-18 [52]. The phenomenon is determined by the modification effect of PPA and the degradation of high temperatures.

**Figure 7:**
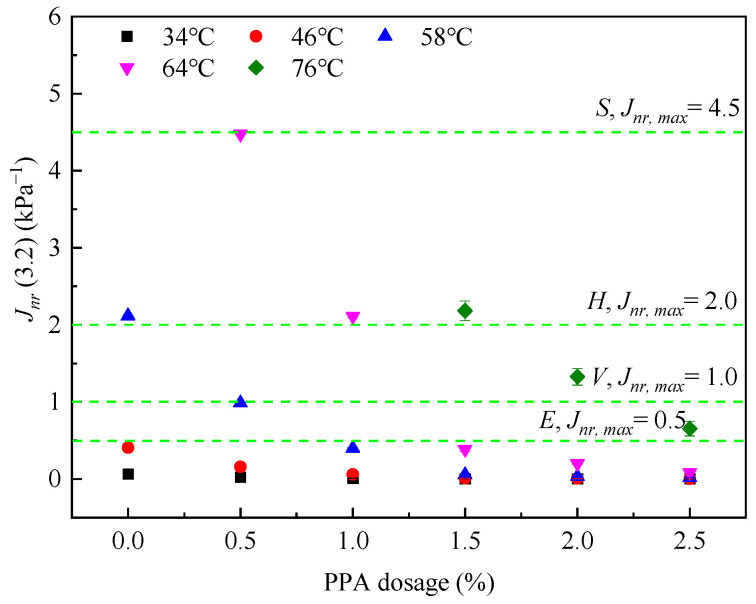
Appropriate traffic levels for PPA−modified asphalt binders.

Enlarge Figure 4d and plot recommended values of $J_{nr}\left(3.2\right)$ (see Table 2) in Figure 7 to determine the applicable traffic levels for PPA-modified asphalt binders.

As shown in Figure 7, six kinds of asphalt binders can meet the requirements of “E” traffic levels at 34 °C and 46 °C; however, these service temperatures are not common in practice. Increasing temperatures will cause traffic levels to change from “E” to “S”, even exceeding the limit of 4.5 kPa^−1^. Combining Figure 6b and Figure 7, the application traffic levels of PPA-modified asphalt binders are shown in Table 3. It is recommended that the designed application temperature of PPA-modified asphalt binders should not be higher than 58 °C. 

### 3.2. Simulation of Creep and Recovery

#### 3.2.1. Creep Behavior

**Figure 8:**
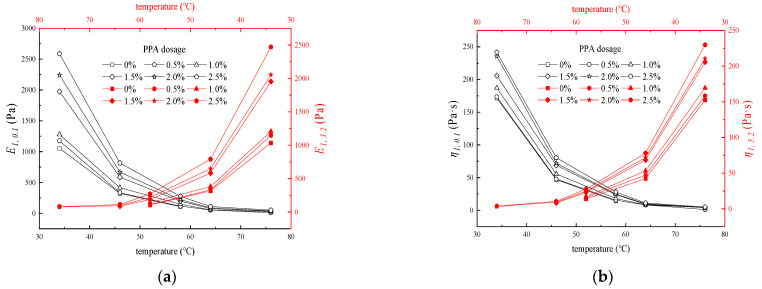

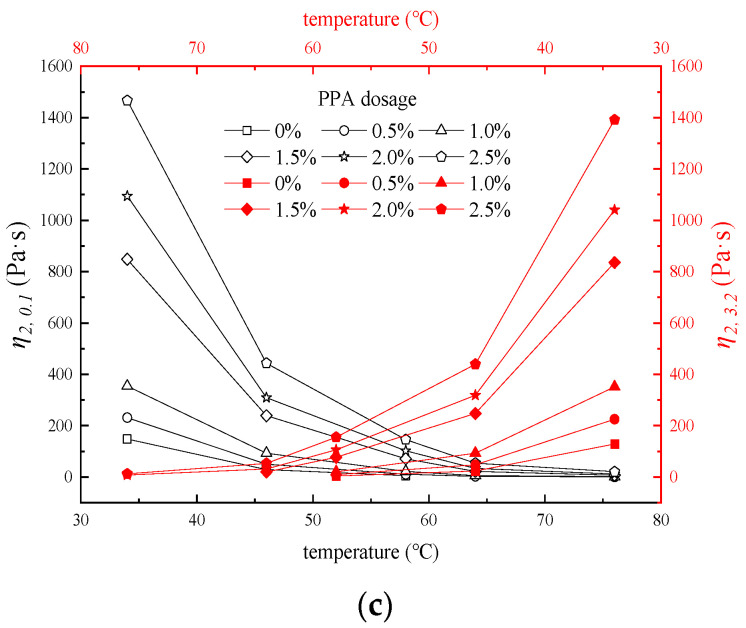
Fitting results at different temperatures and PPA dosage (**a**) $E_{1}$ under different conditions, (**b**) $\eta_{1}$ under different conditions, and (**c**)$\;\eta_{2}$ under different conditions.

The fitting results of the three-element model at different temperatures, PPA dosages, and stress levels are shown in Figure 8.

The three−element model can be used to describe the creep behavior, and the correlation coefficient is all above 0.99. However, fitting results lose the actual physical meaning (where $E_{1}$ has a negative value) at 64 °C and 76 °C, corresponding to the missing points in Figure 8. This may be due to the fact that the elastic component of asphalt binders is reduced under high temperatures, and it is more inclined to viscous fluid.

As shown in Figure 8, $E_{1}$*,* $\eta_{1}$ and $\eta_{2}$ at 0.1 kPa and 3.2 kPa all show a downward trend with the increasing temperature. Curves go down sharply and then show a gentle trend, where 58 °C is the inflection point. In particular, there is only a slight change when the temperature is higher than 64 °C. Meanwhile, increasing PPA dosage can delay the adverse effects of temperature on the deformation properties to some extent. However, the effects are gradually weakened with the increasing temperature, proved by the almost coincident curves at 64 °C and 76 °C. As can be seen from Figure 8, parameters are hardly affected by stress.

In addition, the steady−state creep rate (K) is closely related to the permanent deformation of the material [31]. In this investigation, K was considered to depend only on $\eta_{2}$ according to the three−element model:

(11) $$K=\sigma_{0}/\eta_{2}$$

**Figure 9:**
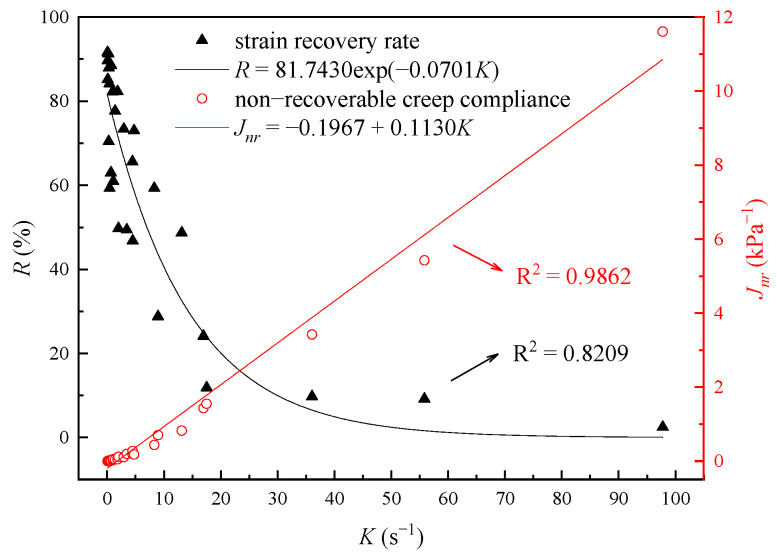
Relationship between K and R, $J_{nr}$.

The relationships between K and deformation parameters are shown in Figure 9.

As shown in Figure 9, R drops exponentially with the increasing K while $J_{nr}$ increases linearly. The quantitative relationship between them can be described by Equations (12) and (13):

(12) $$R=81.7430\times \;\exp\left(-0.0701K\right)$$

(13) $$J_{nr}=-0.1967+0.1130K$$

where the correlation coefficients (R^2^) are 0.8209 and 0.9862, respectively.

It is clear from the R^2^ that there is a better correlation between $J_{nr}$ and *K*. The unrecoverable deformation is greatly affected by the product of K and steady-state creep time. Thus, K is indirectly correlated to R, but directly correlated to $J_{nr}$, explaining the difference in the correlation coefficients. Less residual deformation will occur at the same time when K is smaller, which is beneficial to the high-temperature deformation resistance of asphalt binders. Adding a proper amount of PPA is a good choice.

It is possible to explain the change of rheological parameters in terms of colloidal structure [59,60]. The asphalt binder is a colloidal material with a stable structure, in which asphaltene is surrounded by resin as a dispersed phase disperses in light components [58]. Elastic components may be more closely related to heavy components. The elevated temperature leads to the degradation of the dispersed phase, while the solubility of light components (such as saturates and aromatics) increases [61]. In this process, the elastic and viscous parameters will decrease, and the asphalt binder thus gradually develops into viscous fluid material. As mentioned above, PPA can supplement a portion of the degraded dispersed phase, thereby delaying the process above.

#### 3.2.2. Recovery Behavior

**Figure 10:**
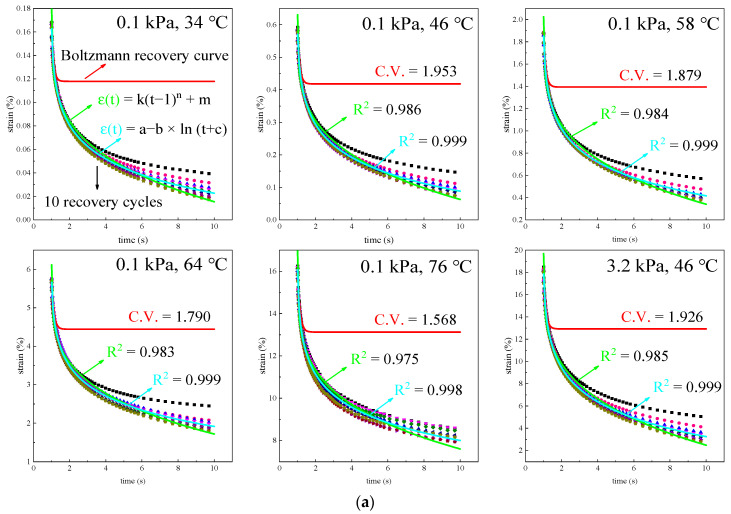

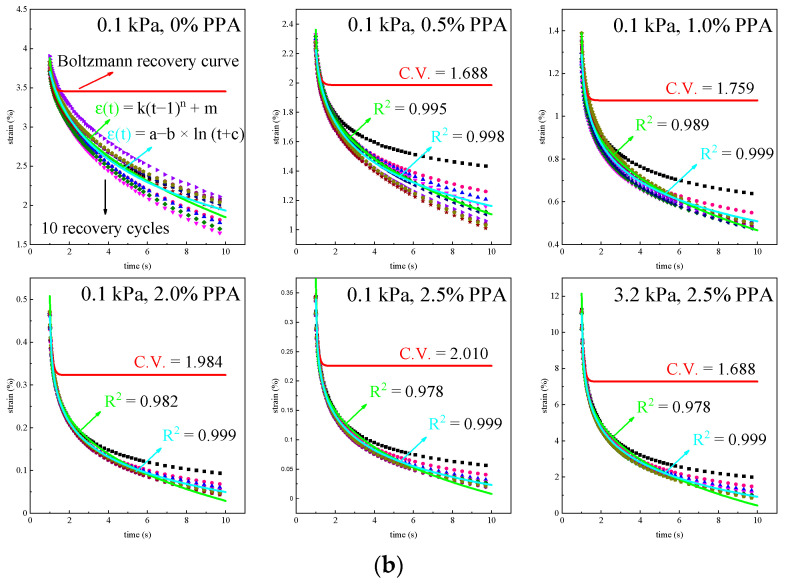
The actual recovery curves and the predicted curves: (**a**) 1.5% PPA at different temperatures; (**b**) different PPA dosages at 46 °C.

The recovery behavior of PPA-modified asphalt binders under different conditions is shown in Figure 10.

If the asphalt binders exhibit linear creep behavior, the Boltzmann superposition principle is applicable. Bring the fitting results of $E_{1}$*,* $\eta_{1}$ and $\eta_{2}$ in the creep stage into Equation (8) to obtain the red predicted curves in Figure 10. It can be clearly observed that the superposition principle can only predict the recovery behavior from 1 s to 2 s, and the asphalt binders have nonlinear creep behavior. To analyze the nonlinear creep behavior quantitatively, Equation (8) is used to fit the actual recovery curves, and parameters $E{}^{\prime }$, $\eta_{1}{}^{\prime }$ and $\eta_{2}{}^{\prime }$ are obtained in Table 4.

As can be seen from Table 4, the influences of test conditions on the parameters are consistent with the creep stage, but the correlation coefficient dropped significantly. The coefficient of variation (C.V.) in the table represents the difference in parameters between the creep stage and the recovery stage. Obviously, asphalt binders will exhibit more pronounced nonlinear creep behavior if more PPA is added, while temperature has the opposite effect. Further, the effect of stress on nonlinear creep behavior is relatively weak.

**Figure 11:**
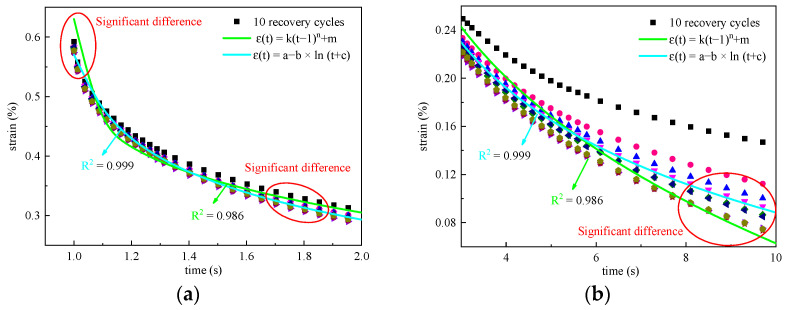
Enlarged view of recovery curves (1.5% PPA at 0.1 kPa and 46 °C): (**a**) Recovery curves for first 2 s; (**b**) recovery curves for 3 s to 10 s.

To describe the recovery behavior more accurately, two empirical models, the power–law model ($\varepsilon \left(t\right)=k\cdot {\left(t-1\right)}^{n}+m$) and Logarithm model ($\varepsilon \left(t\right)=a-b\cdot \ln\left(t+c\right)$), are used for simulation, as shown in Figure 10. It can be seen that both models have good simulation effects, and the correlation coefficients are above 0.97 and 0.99, respectively. The subtle difference between the simulation effects of the two models is shown in Figure 11.

**Figure 12:**
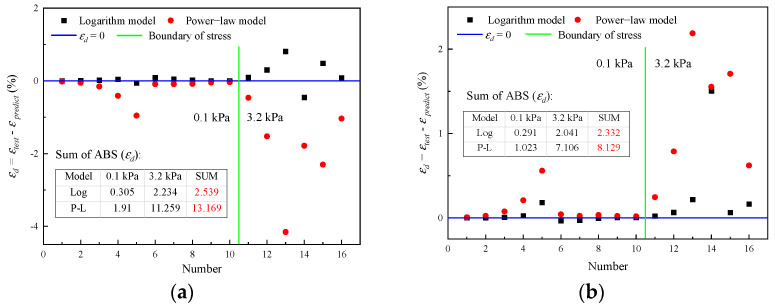
Comparison of prediction effects for initial strain and residual strain: (**a**) Prediction of $\varepsilon_{c}$; (**b**) prediction of $\varepsilon_{r}$.

As shown in Figure 11, the differences are mainly reflected in the initial and final stages of recovery. Compared with the test value, the predicted value of the power–law model for $\varepsilon_{c}$ is too large, while is too small for $\varepsilon_{r}$. Further, the recovery rate changes too sharply. In comparison, the prediction effect of the logarithm model is more acceptable. To confirm this, the difference between the predicted value and the measured value is calculated by $\varepsilon_{d}=\varepsilon_{test}-\varepsilon_{predict}$, as shown in Figure 12.

As can be seen from Figure 12, the prediction effect of the two models will become worse at 3.2 kPa. The total difference shows that, no matter for $\varepsilon_{c}$ or $\varepsilon_{r}$, logarithm model has a better prediction effect. It can be observed in Figure 12b that a residual strain that is smaller than the actual situation will be predicted by both models. Overall, it is acceptable to choose the logarithmic model to simulate recovery behavior.

### 3.3. Energy Analysis

**Figure 13:**
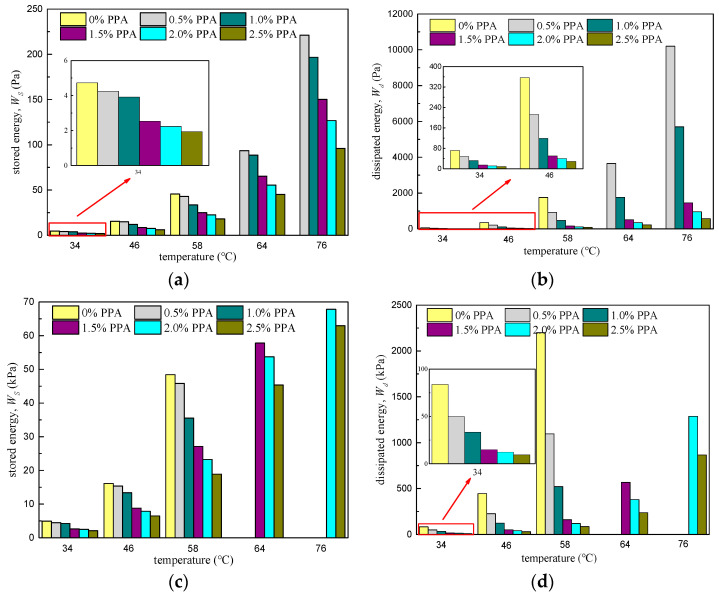
Calculation results of $W_{s}\left(t\right)$ and $W_{d}\left(t\right):$ (**a**) Stored energy $W_{s}\left(t\right)$ at 0.1 kPa; (**b**) dissipated energy $W_{d}\left(t\right)$ at 0.1 kPa; (**c**) stored energy $W_{s}\left(t\right)$ at 3.2 kPa; (**d**) dissipated energy $W_{d}\left(t\right)$ at 3.2 kPa.

Stored energy $W_{s}\left(t\right)$ and dissipated energy $W_{d}\left(t\right)$ are calculated by Equations (5) and (6), respectively, and the results are shown in Figure 13.

It can be seen from Figure 13 that both $W_{s}\left(t\right)$ and $W_{d}\left(t\right)$ rise sharply with the increasing temperature, and drop obviously after more PPA was added. The influence of temperature on energy parameters is more significant than that of PPA dosage. As proof, $W_{s}\left(t\right)$ and $W_{d}\left(t\right)$ decreased by 60.3% and 91.9% when PPA dosage was raised from 0% to 2.5% at 46 °C, and the parameters successively increased by 5834.0% and 10,088.5% from 34 °C to 76 °C for 1.5% PPA.

**Figure 14:**
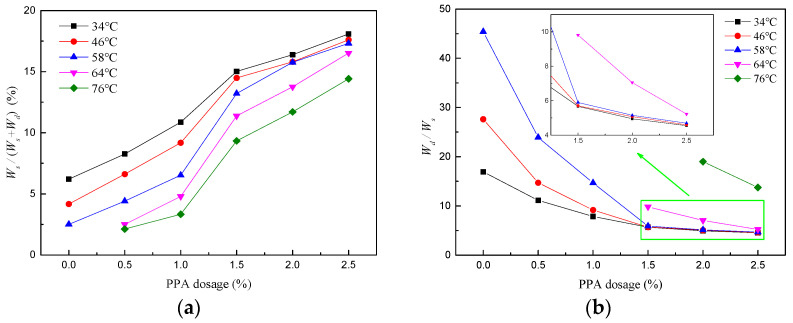
Energy ratio under different test conditions: (**a**) Percentage of stored energy at 0.1 kPa; (**b**) dissipation energy ratios at 3.2 kPa.

The relative values of $W_{s}\left(t\right)$ and $W_{d}\left(t\right)$ under the same test conditions can reflect the ratio of viscous components and elastic components in asphalt binders. Therefore, the percentage of stored energy [$W_{s}\left(t\right)/\left(W_{s}\left(t\right)+W_{d}\left(t\right)\right)*100\%$] and dissipation energy ratios [$W_{d}\left(t\right)/W_{s}\left(t\right)$] are calculated, as shown in Figure 14. The former characterizes deformation recovery ability, while the latter is mainly correlated to unrecoverable deformation.

As detailed in Figure 14a, the percentage of stored energy goes up appreciably with the increase in PPA dosage, which corresponds to a decline in the dissipation energy ratios in Figure 14b. As discussed previously, PPA can promote asphalt binders to form a more stable colloidal structure and strengthen the interaction between asphalt components. Thus, more work is converted into molecular potential energy to be stored. Then the energy is released after the stress is removed, providing energy for deformation recovery. This is also the reason why the deformation recovery ability of asphalt binders is improved with the increasing PPA dosage. Meanwhile, as can be seen from the slope of curves in Figure 14, the variation rate of the energy ratio gradually declines after PPA dosage exceeds 1.5%.

### 3.4. Analysis of Creep–Recovery Mechanism

#### 3.4.1. Rheological Behavior Analysis

**Figure 15:**
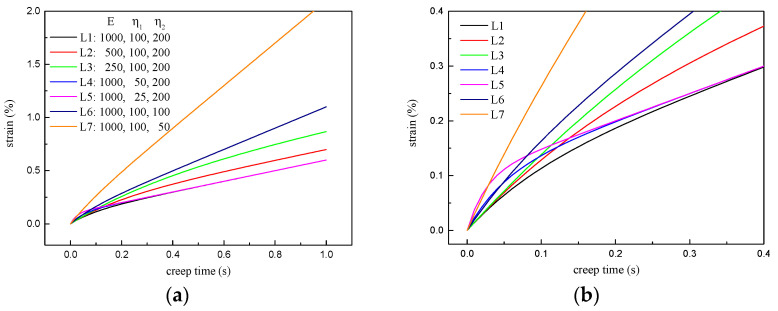

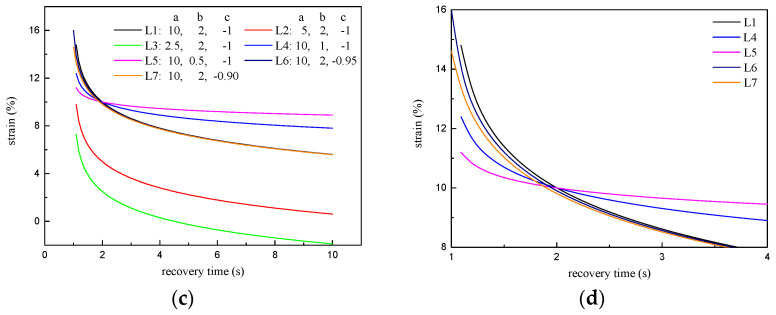
Response of creep−recovery curves to model parameters: (**a**) Three−element model for creep stage; (**b**) enlarged view of figure; (**a**,**c**) logarithm model for recovery stage; (**d**) enlarged view of figure (**c**).

The rheological model is an idealized analysis method for the mechanical and deformation properties of materials [35,62]. The variation of model parameters can theoretically reflect the changes in the properties of the material. The response of creep–recovery behavior to model parameters is shown in Figure 15.

As shown in Figure 15a,b, $E_{1}$ mainly affects the total strain and the initial creep rate but hardly affects K. The increase in the elastic component reduces the initial creep rate, thus producing a smaller total strain. This is the reason why PPA can improve the deformation performance of asphalt binders. Reduced $\eta_{1}$ leads to a greater initial creep rate and earlier time to enter the steady-state creep stage; however, they have an effect on K and total strain. $\eta_{2}$ has a significant effect on initial creep rate, total strain, and K. The three indicators will increase sharply as $\eta_{2}$ decreases. This also explains the attenuation modification effects of PPA on asphalt binders at high temperatures. In summary, $E_{1}$ and $\eta_{1}$ characterize the delay elastic deformation of asphalt binders, and play a decisive role in initial creep rate, while steady creep stage is determined by $\eta_{2}$.

It can be seen from Figure 15c,d, a in the logarithm model will affect the initial recovery strain and residual strain but will not change the recovery rate. When only b changes, the recovery curves will intersect at a certain moment. Before this time point, a larger b means a greater strain, and it is the opposite when asphalt binders continue to recover. The change of c will only affect the recovery rate within a very short time. Since the logarithm model is only an empirical model, it can only be used to predict recovery behavior rather than a theoretical explanation.

#### 3.4.2. Correlation between Deformation and Energy Parameters

**Figure 16:**
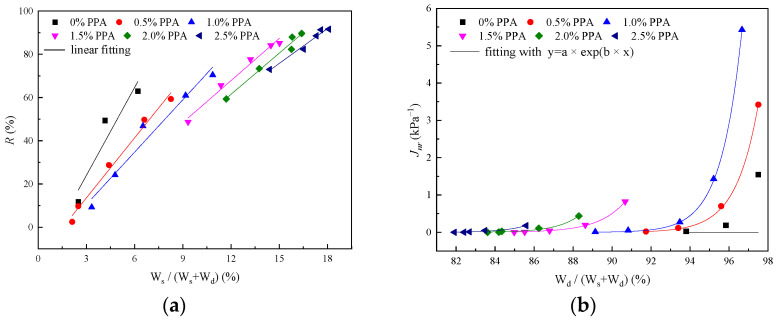
Correlation between deformation parameters and energy parameters: (**a**) Relationship between R and $\frac{W_{S}}{W_{S}+W_{d}};$ (**b**) relationship between $J_{nr}$ and $\frac{W_{d}}{W_{S}+W_{d}}$.

The creep–recovery phenomenon of asphalt binders is a macroscopic manifestation of energy conversion and dissipation. In the creep process, $E_{1}$ stores a portion of the energy to form stored energy, $\eta_{1}$ and $\eta_{2}$ are viscous components, and the remaining energy is consumed. As the applied force is removed, part of the stored energy is directly released, and the other part is used for deformation recovery. Figure 16 shows the relationships between deformation parameters and energy parameters.

As shown in Figure 16, there may be a linear relationship between R and the storage energy ratio while $J_{nr}$. The dissipation energy ratio might be in an exponential relationship, and the fitting results are shown in Table 5.

As shown in Table 5, the correlation coefficient is above 0.95 (except for the neat asphalt binder), implying a good linear correlation between R and $W_{S}/\left(W_{S}+W_{d}\right)$ and a good exponential correlation between $J_{nr}$ and $W_{d}/\left(W_{S}+W_{d}\right)$. With the increase in storage energy ratio, more energy is used for recovery. However, the linear proportional coefficient shows a declining trend as PPA dosage ascends; that is, the increase in R caused by the unit storage energy ratio shows a decreasing trend. This may be caused by the limitation of the improvement effect of PPA.

The exponential correlation between non-recoverable creep compliance and dissipation energy ratio is above 0.97. As shown in Figure 16b, $J_{nr}$ will promote dramatically when the dissipation energy ratio is more than 90%. A higher dissipation energy ratio means an increase in viscous deformation, which usually forms residual deformation, thus $J_{nr}$ raises accordingly. More elastic components in the asphalt binder will form after PPA is added. Hence, the residual deformation dwindles and $J_{nr}$ is relatively stable. Overall, there is a good correlation between deformation parameters and energy parameters, revealing the creep and recovery behavior of PPA-modified asphalt binders in different ways.

## 4. Conclusions and Suggestions

### 4.1. Conclusions

In this work, an important argument for the life of the road pavement was discussed, that is, how the PPA-modified binders answer to the deformation under multiple factors. The influences of temperature, PPA dosage, and stress on the creep–recovery behavior were tested by MSCR test. The occurrence, development, and recovery of deformation behaviors were analyzed by deformation parameters, rheological theories, energy changes, and simulation methods. The main conclusions are as follows:

The elastic component of the asphalt can be increased by PPA, which contributes to its high-temperature performance. Taking into account the deformation recovery and workability of PPA-modified asphalt, the recommended dosage of PPA is 1.5%.

The creep behavior of PPA-modified asphalt binders can be accurately characterized by Three- element model, while the recovery behavior is well simulated by the Logarithmic model. In this investigation, PPA-modified asphalt binders exhibit nonlinear creep behavior.

Steady-state creep rate (K) can be simplified as $\sigma_{0}/\eta_{2}$, which is indirectly related to R, and is directly related to $J_{nr}$. Similarly, stored energy directly affects R, and dissipated energy indirectly affects $J_{nr}$.

The energy storage capacity of asphalt binders can be improved by the changed colloid structure caused by PPA, thus improving the deformation recovery ability of asphalt binders.

### 4.2. Limitations and Suggestions

Despite the fact that PPA can significantly improve the deformation recovery of the asphalt binder, its negative impact on workability cannot be ignored. In particular, the viscosity of the modified asphalt binder at 135 °C exceeds 3.0 Pa·s when PPA is added more than 2.0%, which is beyond the recommendations of the SHRP guidelines. In addition, some of the findings in this investigation are closely related to the nature of the neat asphalt binder (e.g., traffic levels), and more types of neat asphalt binder are proposed to be investigated. In order to promote the application of PPA-modified asphalt binders, a more comprehensive investigation of its road performance should be given in future studies.

## Figures and Tables

**Table 1:** Basic physical properties of six kinds of asphalt binders.

PPA Dosage (%)	Penetration (25 °C, 5 s, 100 g)/0.1 mm	Softening Point (R&B)/°C	Penetration Index	135 °C Viscosity/Pa·s
0	89.9	45.9	−0.698	0.435
0.5	63.3	51.0	0.909	0.665
1.0	53.1	55.9	1.905	0.995
1.5	45.7	64.7	2.931	2.104
2.0	42.6	75.2	3.129	4.745
2.5	38.3	76.6	3.857	over 5.000
Test Methods	ASTM D5 [48]	ASTM D36 [49]	JTG E20 T0604 [47]	ASTM D4402 [50]

**Table 2:** $J_{nr}\left(3.2\right)$ and $J_{nrdiff}$ for different traffic levels.

Traffic Levels	S	H	V	E
$J_{nr}\left(3.2\right)$/kPa^−1^, max	4.5	2.0	1.0	0.5
$J_{nrdiff}$/%, max	75			

**Table 3:** Suggested traffic levels for PPA-modified asphalt binders.

Conditions	0% PPA	0.5% PPA	1.0% PPA	1.5% PPA	2.0% PPA	2.5% PPA
34 °C	$J_{nrdiff}$ > 75%	E	E	E	E	E
46 °C						
58 °C	S	V	E	E	E	E
64 °C	$J_{nr}\left(3.2\right)$>	S	S	$J_{nrdiff}$ > 75%		
76 °C	4.5 kPa^−1^	$J_{nr}\left(3.2\right)$ > 4.5 kPa^−1^				

**Table 4:** Fitting results of Equation (8) to recovery stage.

Test Conditions			Model Parameters			R^2^	Coefficient of Variation
Stress	PPA	Temperature	$E{}^{\prime }\;(\mathrm{Pa})$	$\eta_{1}{}^{\prime }\;(\mathrm{Pa}\cdot s)$	$\eta_{2}{}^{\prime }\;(\mathrm{Pa}\cdot s)$		
0.1 kPa	1.5%	34 °C	700.23	604.01	2559.86	0.951	2.011
		46 °C	198.16	176.72	688.80	0.950	1.953
		58 °C	67.38	59.07	170.27	0.951	1.879
		64 °C	25.91	22.52	42.31	0.951	1.790
		76 °C	12.91	10.10	11.13	0.952	1.568
	0%	46 °C	36.69	53.02	46.46	0.959	1.633
	0.5%		74.68	84.54	77.78	0.951	1.688
	1.0%		113.27	111.97	166.13	0.954	1.759
	2.0%		247.52	203.52	1015.48	0.949	1.984
	2.5%		339.28	259.23	1656.79	0.947	2.010
3.2 kPa	1.5%	34 °C	686.46	595.67	2542.87	0.950	2.011
		46 °C	209.47	186.12	644.35	0.951	1.926
		58 °C	76.59	68.83	145.56	0.952	1.800
	0%	46 °C	317.96	146.63	24.51	0.978	0.832
	1.0%		127.43	121.23	146.07	0.955	1.664
	2.5%		337.32	259.53	1522.45	0.948	1.997

**Table 5:** Fitting results between deformation parameters and energy parameters.

PPA Dosage (%)	$R=\alpha \times \frac{W_{S}}{W_{S}+W_{d}}+\beta$			$J_{nr}=\gamma \times exp\left(\lambda \times \frac{W_{d}}{W_{S}+W_{d}}\right)$		
	α	β	*R* ^2^	γ	λ	*R* ^2^
0	13.5424	−16.6829	0.79802	—	—	—
0.5	9.2558	−14.2327	0.98299	2.53 × 10^−37^	0.87693	0.99924
1.0	8.0831	−13.7822	0.95308	2.29 × 10^−39^	0.93796	0.99985
1.5	6.4850	−9.9931	0.97756	6.49 × 10^−30^	0.73913	0.99886
2.0	6.3442	−14.6478	0.96714	8.52 × 10^−34^	0.85319	0.98913
2.5	5.3738	−4.8488	0.96778	1.17 × 10^−33^	0.866	0.97179

## Data Availability

The datasets analyzed during the current study are available from the corresponding author upon reasonable request.
